# Relationship between Cariogenic Bacteria and pH of Dental Plaque at Margin of Fixed Prostheses

**DOI:** 10.1155/2012/452108

**Published:** 2012-01-11

**Authors:** Junko Tanaka, Norio Mukai, Muto Tanaka, Masahiro Tanaka

**Affiliations:** Department of Fixed Prosthodontics and Occlusion, Osaka Dental University, 1-5-17, Otemae, Chuo-ku, Osaka 540-0008, Japan

## Abstract

*Objective*. The purpose of this study was to investigate whether teeth that have undergone prosthetic restoration are under conditions that promote caries recurrence. *Methods*. The subjects were 20 dentate adults with both a healthy tooth and an affected tooth entirely covered with a complete cast crown in the molar regions of the same arch. The pH was measured in plaque adhering to the margin of the tooth covered with a complete cast crown and adhering to the cervicobuccal area of the natural tooth. In addition, the numbers of cariogenic bacteria (mutans streptococci and lactobacilli) were measured employing the saliva test. The relationships between the number of cariogenic bacteria and plaque pH of the natural tooth and between the number of cariogenic bacteria and plaque pH of the tooth covered with a complete cast crown were investigated. *Results*. The plaque pH of the tooth covered with a complete cast crown decreased as the numbers of SM and LB increased. The natural tooth were also influenced by the number of SM. *Conclusion*. Secondary caries are likely to develop from the marginal region of the crown in the oral cavity with a high caries risk unless a preventive program is prepared and the oral environment is improved following the program.

## 1. Introduction

Secondary caries from the margin of fixed prostheses is often noted in daily clinical cases, occasionally leading to tooth extraction due to the development of subgingival carious cavities. The secondary caries is considered to occur from various factors, such as the poorly fitting margin, and plaque accumulation.

Khöler and Hager reported that mutans streptococci adhere to the margins of the restorations early after the cementing in high-risk group regarding the cariogenic bacteria counts [1]. When fixed prostheses are left in the high caries activities, the possibility of the incidence of secondary caries becomes high.

When the incidence of caries was investigated in 19-year-old Japanese and Swedish subjects, the prevalence of caries and DMFT were higher in Japanese, and this may have resulted from differences in the preventive strategy for children and young people [2, 3]. Prevention of the development and progression of caries since infancy reduces the risk of caries in adulthood and prevents tooth loss. Therefore, it is necessary to understand the importance of “treatment of process” of caries incidence.

The “treatment of process” means the performance of patient risk evaluation and instruction and treatment according to prevention programs produced based on the risk evaluation. This treatment target is chiefly the process of infection and decalcification before the incidence of dental cavities. On the other hand, conventional restorative treatment of dental cavities is regarded as “treatment of results”. Even if the restorative treatment is appropriately performed, the oral environment does not improve. The secondary caries will occur from the margin of the restorations when the oral environment does not improve. After the result of repetitive retreatment, multiple teeth will be lost. This is because the “process” during which prostheses are cemented and appropriate treatment is not performed.

Preceding studies clarified that caries development is strongly associated with caries factors, particularly microbial factors [4]. However, in performing “treatment of process” for the prevention of secondary caries, the influence of the SM and LB counts on the pH value of plaque in the crown margin has not been clarified.

In this study, as the first step of the “treatment of process”, regarding caries risk, the relationship between the cariogenic bacteria counts and plaque pH was evaluated.

## 2. Material and Method

### 2.1. Study Design and Subjects

Among middle-aged and elderly patients who visited our hospital, 20 dentate (7 males and 13 females, mean age: 51.1 ± 12.1 years, number of remaining teeth: 25.8 ± 2.1) were selected as the subjects. They were performed using only fixed prostheses. In the oral situations of the subjects, both natural teeth and fixed prostheses were present in the molar area on the contralateral teeth in the same arch.

The subjects' teeth consisted of 41 healthy teeth without caries or restorations (natural tooth group) and 69 molars in which silver-palladium gold-alloy complete cast crown was cemented with a good fit (crown group). The adaptation of the complete cast crown was examined using the explorer [5].

**Figure 1:**
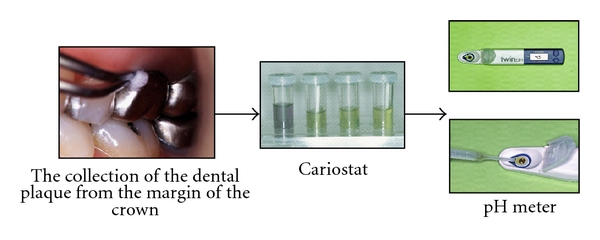
Measurement of the pH values of dental plaque.

The Cariostat (Dentsply-Sankin, Tokyo, Japan) was used for measuring the pH values of dental plaque. The dental plaque was collected one hour or longer after completing brushing. The cervicobuccal area of subject teeth was wiped with sterilized swabs about ten times. They were immediately put into the culture solution. The culture solution was cultivated in an incubator at 37 degrees for 48 hours. Then, the pH values were measured using a pH meter (B-212, Horiba, Tokyo, Japan) to obtain the pH values of the dental plaque (Figure 1).

Regarding measurement of the cariogenic bacteria counts, such as mutans streptococci (SM) and lactobacilli (LB), stimulated saliva was collected using the spit-out method after chewing 1 gram of paraffin wax for 5 minutes. The collection of saliva was performed between nine thirty and eleven thirty in the morning more than 1 hour after breakfast.

The stimulated saliva was cultivated using a simplified culture kit (Dentocult SM, LB, Diagnostics Co.). After cultivation, the number of SM and LB was measured, comparing with an attached model chart. The results were divided into 4 classes (Table 1).

This study protocol was screened and approved on its ethical acceptability by the Committee on Experimental Research on Human of Osaka Dental University.

### 2.2. Statistical Analysis

We compared the pH values of dental plaque and the SM and LB counts using one-way ANOVA. Regarding combinations in which significant differences were noted, a multiple comparison test was performed using Fisher's PLSD method (*P* < 0.05).

## 3. Results

**Figure 2:**
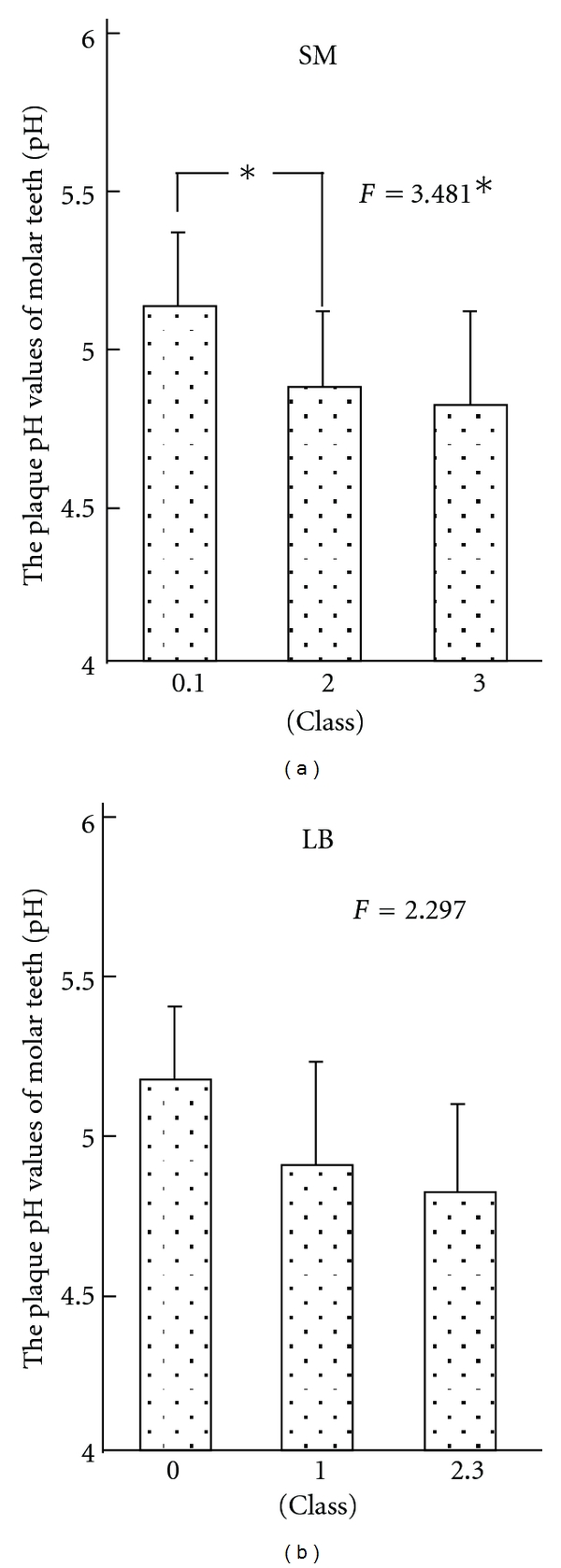
Relationship between the plaque pH values of molar teeth and oral environmental factor in the natural teeth group.

Figure 2 shows the result in the natural tooth groups.

In the natural tooth group, the pH values of dental plaque became lower with an increase in the SM counts, showing a significant difference. Therefore, a multiple comparison test was performed, and a significant difference was noted between classes 0 and 1 and class 2 in SM. The pH value became lower with an increase in the LB counts. However, no statistically significant difference was observed between the pH values and the LB counts.

**Figure 3:**
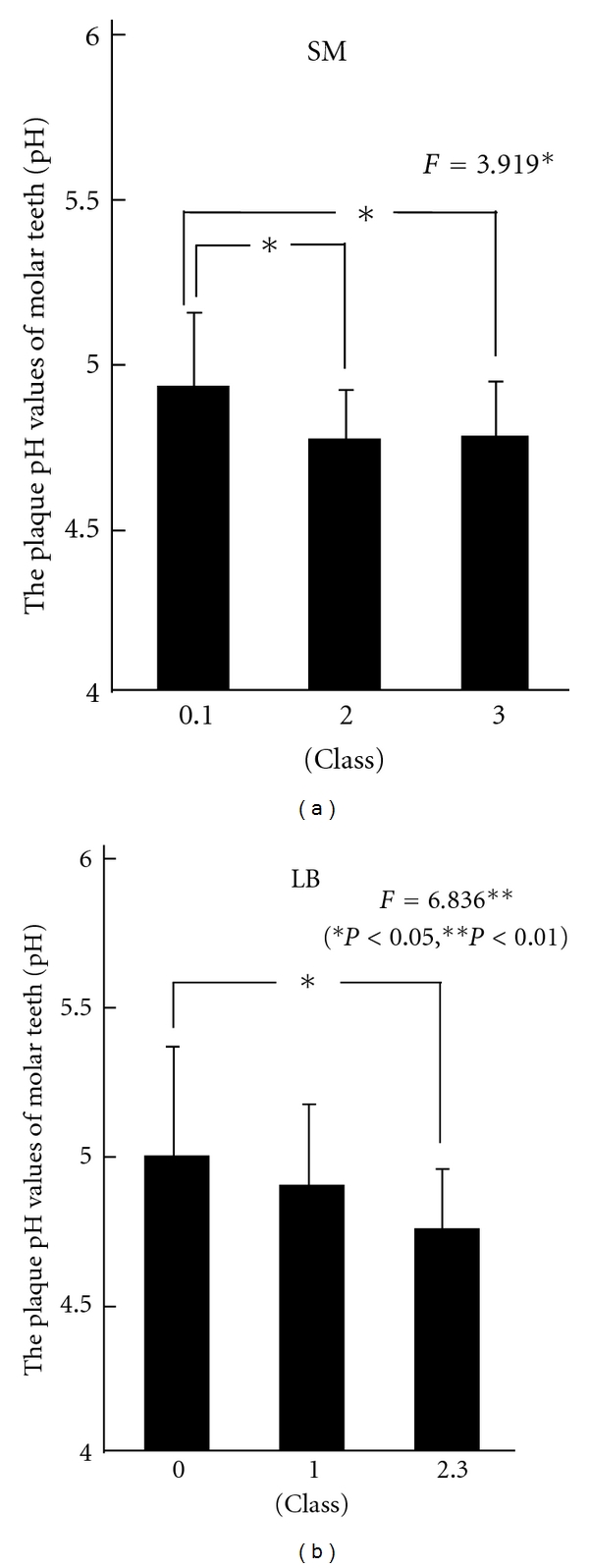
Relationship between the plaque pH values of molar teeth and oral environmental factor in the crown group.

Figure 3 shows the results in the crown groups.

In the crown group, the pH value became lower with an increase in the SM counts, showing a significant difference. Furthermore, the plaque pH values significantly became lower with an increase in the LB counts. As a result of multiple comparison tests, significant differences were between classes 0 and 1 and class 2, and between classes 0 and 1 and class 3 in SM, showing decreases in the pH value with increases in SM as a caries risk. Regarding LB, a significant difference was noted between class 0 and classes 2 and 3. The pH values of dental plaque were lower with increases in the LB counts.

## 4. Discussion

### 4.1. Method

#### 4.1.1. Subjects

The subjects were selected; only fixed restorative appliances were inserted. Our previous study revealed that the cariogenic bacteria counts were high in patients wearing removable dentures, in which the adhesion of bacteria to the dentures wearers was considered [6]. Therefore, removable denture wearers were not selected as the subjects in this study.

#### 4.1.2. Materials

Dentocult was used to investigate the oral environment concerning caries. Its usefulness has been reported by clinical studies in many countries, and an epidemiological survey by the WHO, [7, 8] including not only measurement of the flow rate and buffer capacity of stimulated saliva and cariogenic SM and LB counts but also microbiological analysis has been performed.

The Cariostat used in the experiments is widely used in caries activity tests [9, 10]. With the Cariostat TM method, the dental plaque is put into a test solution containing a high concentration of sucrose, and acid is generated as the plaque constituting bacteria grow. The caries activity is evaluated based on the changes in colors of the pH indicator in the test solution with the reduced pH using acid. Okazaki et al. [11] researched the reproducibility of the Cariostat method.

The method of Yamaga et al. [12] where the pH values of cultures were measured with a pH meter was adopted in order to make this study accurate.

### 4.2. Results

Previous studies confirmed that there is no significant difference in the pH of the plaque adhering to the cervicobuccal area of natural molars between the contralateral teeth in the same arch [3]. Therefore, we considered that there is no difference in the condition of tooth brushing and flow of saliva between the contralateral teeth in the same arch.

In the natural tooth group, the plaque pH has become lower with an increase in the SM counts. The number of SM tended to increase as the plaque pH in the tooth neck decreased, but a significant difference was noted only between the groups with low and medium SM risks. This may have been due to variation of the plaque pH among subjects with a high SM risk. SM is a cariogenic bacterium which adheres even to the smooth surfaces of teeth. Decalcification of cementum is also likely to progress in natural teeth when the root surface is exposed.

On the other hand, the plaque pH was also lowered with the LB counts. However, no significant difference was observed. However, a study involving adults reported that the number of lactobacillus was significantly associated with caries of the smooth and adjacent surfaces and, particularly, the relationships with prostheses and secondary caries, for which further investigation is necessary [13].

 In the crown group, it was revealed that the pH of the plaque adhering to the marginal area was influenced by both SM and LB counts. The plaque pH in the marginal region of the crown decreased as the number of cariogenic bacteria increased. The plaque pH was lower than that in the natural tooth group, suggesting an influence of the number of bacteria. LB is frequently detected in uneven regions, such as pits, fissures, and carious cavities. It was reported that the numbers of SM and LB increased in the oral cavity after a fixed orthodontic appliance was applied, particularly the number of LB [14], suggesting that LB readily adheres to the marginal region of the crown. It is likely that secondary caries develop soon unless the risk is reduced, even though a crown with a good fit is applied.

## 5. Conclusions

To achieve the “treatment of process” of secondary caries, the relationship between the cariogenic bacteria counts and plaque pH in natural teeth and fixed prostheses was investigated. As a result, it was revealed that the plaque adhering to the natural teeth was influenced only by the SM counts, whereas the plaque adhering to the fixed prostheses was influenced by both the SM and LB counts.

These findings suggest that it is important to decrease the cariogenic bacteria counts from the perspective of caries prevention. In particular, to prevent secondary caries in the margin of prosthetic appliances, it is important to decrease the LB counts.

## Figures and Tables

**Table 1:** Classification of oral environmental factors.

Oral environmental factors	Level			
	0	1	2	3
mutans streptococci (CFU/ml)	<1 × 10^5^		1 × 10^5^ ~ 1 × 10^6^	>1 × 10^6^
lactobacilli (CFU/ml)	≦1 × 10^3^	1 × 10^4^	1 × 10^5^	≧1 × 10^6^

CFU: colony forming units.

## References

[1] Köhler B, Hager B (1993). Influence of salivary levels of mutans streptococci on colonization of crown margins: a longitudinal study. *The Journal of Prosthetic Dentistry*.

[2] Wang NJ, Källestål C, Petersen PE, Arnadottir IB (1998). Caries preventive services for children and adolescents in Denmark, Iceland, Norway and Sweden: strategies and resource allocation. *Community Dentistry and Oral Epidemiology*.

[3] Kallestål C, Wang NJ, Petersen PE, Arnadottir IB (1999). Caries-preventive methods used for children and adolescents in Denmark, Iceland, Norway and Sweden. *Community Dentistry and Oral Epidemiology*.

[4] Milgrom P, Riedy CA, Weinstein P, Tanner ACR, Manibusan L, Brass J (2000). Dental caries and its relationship to bacterial infection, hypoplasia, diet, and oral hygiene in 6- to 36-month-old children. *Community Dentistry and Oral Epidemiology*.

[5] Tanaka J, Kawazoe T, Iwayama Y (2005). Influence of the fit of fixed prostheses margins on the pH of dental plaque. *Prosthodontic Research and Practice*.

[6] Tanaka J, Nishikawa M, Tatsuta M (2003). The differences of oral environment between the elderly wearing fixed and removable prostheses. *Journal of Osaka Dental University*.

[7] Jensen B, Bratthall D (1989). A new method for the estimation of mutans streptococci in human saliva. *Journal of Dental Research*.

[8] Larmas M (1992). Saliva and dental caries: diagnostic tests for normal dental practice. *International dental journal*.

[9] Shimono T, Mizuno J, Nonomura E (1976). Studies of a simple new colorimetric method (Cariostat) for determining the carious activity: (1) Comparison with an improved Snyder test. *The Japanese Journal of Pediatric Dentistry*.

[10] Nishimura M, Chen HJ, Docor R (1994). The relationship between a caries activity test (Cariostat) and plate colony counts of mutans streptococci in human dental plaque. *International journal of Japanese Society of Pediatric Dentistry*.

[11] Okazaki Y, Tanaka K, Bazarrgchaa T (1998). A study of the validity of the caries activity test (Cariostat) part 1. *The Japanese Journal of Pediatric Dentistry*.

[12] Yamaga T, Komoda Y, Itosaka N (1995). Root surface caries and caries activity of dental plaque. *The Journal of the Japan Prosthodontic Society*.

[13] Nishikawara F, Katsumura S, Ando A (2006). Correlation of cariogenic bacteria and dental caries in adults. *Journal of oral science*.

[14] Hamasaki T, Awano S, Konoo T (2009). Utility of a simplified caries risk test for patients with fixed orthodontic appliances. *Journal of Dental Health*.

